# Use of Machine Learning Techniques for Case-Detection of Varicella Zoster Using Routinely Collected Textual Ambulatory Records: Pilot Observational Study

**DOI:** 10.2196/14330

**Published:** 2020-05-05

**Authors:** Corrado Lanera, Paola Berchialla, Ileana Baldi, Giulia Lorenzoni, Lara Tramontan, Antonio Scamarcia, Luigi Cantarutti, Carlo Giaquinto, Dario Gregori

**Affiliations:** 1 Department of Cardiac Thoracic Vascular Sciences and Public Health University of Padova Unit of Biostatistics, Epidemiology and Public Health Padova Italy; 2 Department of Clinical and Biological Science University of Turin Torino Italy; 3 Arsenàl.IT Treviso Italy; 4 Società Servizi Telematici, Pedianet Padova Italy; 5 Department of Women's and Children's Health University of Padova Padova Italy

**Keywords:** machine learning technique, text mining, electronic health report, varicella zoster, pediatric infectious disease

## Abstract

**Background:**

The detection of infectious diseases through the analysis of free text on electronic health reports (EHRs) can provide prompt and accurate background information for the implementation of preventative measures, such as advertising and monitoring the effectiveness of vaccination campaigns.

**Objective:**

The purpose of this paper is to compare machine learning techniques in their application to EHR analysis for disease detection.

**Methods:**

The Pedianet database was used as a data source for a real-world scenario on the identification of cases of varicella. The models’ training and test sets were based on two different Italian regions’ (Veneto and Sicilia) data sets of 7631 patients and 1,230,355 records, and 2347 patients and 569,926 records, respectively, for whom a gold standard of varicella diagnosis was available. Elastic-net regularized generalized linear model (GLMNet), maximum entropy (MAXENT), and LogitBoost (boosting) algorithms were implemented in a supervised environment and 5-fold cross-validated. The document-term matrix generated by the training set involves a dictionary of 1,871,532 tokens. The analysis was conducted on a subset of 29,096 tokens, corresponding to a matrix with no more than a 99% sparsity ratio.

**Results:**

The highest predictive values were achieved through boosting (positive predicative value [PPV] 63.1, 95% CI 42.7-83.5 and negative predicative value [NPV] 98.8, 95% CI 98.3-99.3). GLMNet delivered superior predictive capability compared to MAXENT (PPV 24.5% and NPV 98.3% vs PPV 11.0% and NPV 98.0%). MAXENT and GLMNet predictions weakly agree with each other (agreement coefficient 1 [AC1]=0.60, 95% CI 0.58-0.62), as well as with LogitBoost (MAXENT: AC1=0.64, 95% CI 0.63-0.66 and GLMNet: AC1=0.53, 95% CI 0.51-0.55).

**Conclusions:**

Boosting has demonstrated promising performance in large-scale EHR-based infectious disease identification.

## Introduction

Improving the predictive capability of infectious disease detection at the population level is an important public health issue that can provide the background information necessary for the implementation of effective control strategies, such as advertising and monitoring the effectiveness of vaccination campaigns [1].

The need for fast, cost-effective, and accurate detection of infection rates has been widely investigated in recent literature [2]. Particularly, the combination of increased electronic health report (EHR) implementation in primary care, the growing availability of digital information within the EHR, and the development of data mining techniques offer great promise for accelerating pediatric infectious disease research [3].

Although EHR data are collected prospectively in real time at the point of health care delivery, observational studies intended to retrospectively assess the impact of clinical decisions are likely the most common type of EHR-enabled research [3].

Among the high-impact diseases, the prompt identification of varicella zoster viral infections is of key interest due to the debate around the need and cost-benefit dynamics of a mass-vaccination program for young children [4,5].

Challenges in this context arise from both the unique epidemiological characteristics of varicella zoster with respect to information extraction, such as age-specific consultation rates, seasonality, force of infection, hospitalization rates, and inpatient days [6], and from the way that medical records are organized, often in free-format and uncoded fields [7]. A critical step is to transform this large amount of health care data into knowledge.

Data extraction from free text for disease detection at the individual level can be based on manual, in-depth examinations of individual medical records or, to contain costs and ensure time-tightening and control, by automatic coding. Machine learning techniques (MLTs) are the most commonly used approaches [8] and show good overall performance [9,10]. Nevertheless, few indications are currently available on the most appropriate technique to use, and comparative evidence is still lacking on the performances of each available technique [11] in the field of pediatric infectious disease research.

In recent years, generalized linear model (GLM)-based techniques have been largely used for the text mining of EHRs, both as a technique of choice [12] and as a benchmark [13]. The performance of GLMs, especially multinomial or in the simplest cases logistic regression, has been indicated as unsatisfactory [14] because they are prone to overfitting and are sensitive to outliers. Enhancements to GLMs have been proposed recently in the form of the lasso and elastic-net regularized GLM [15] (GLMNet), multinomial logistic regression (maximum entropy [MAXENT]), and the boosting approach implemented in the LogitBoost algorithm [16] to overcome the limitations of naïve GLMs. Nevertheless, to the best of our knowledge, no comparisons have been made among these techniques to determine to what extent improvements are needed.

The purpose of this study is to make comparisons among enhanced GLM techniques in the setting of automatic disease detection [17]. Particularly, these methods will be assessed on their ability of identifying cases of varicella from a large set of EHRs.

## Methods

### Electronic Medical Record Database

The Italian Pedianet database [18] collects anonymized clinical data from more than 300 pediatricians throughout the country. This database focuses on children 0-14 years of age [19-22] and records the reasons for accessing health care, diagnosis, and clinical details. The sources of those data are primary care records written in Italian, which are filled in by pediatricians with clinical details about diagnosis and prescriptions; they also contain details about the eventual hospitalization and specialist referrals.

For the purpose of this study, we were allowed to access only two subsets of the Pedianet database, corresponding to the data collected between 2004 and 2014 in the Italian regions of Veneto (northern Italy) and Sicilia (South Italy). Since the Veneto region data set was larger, it was considered for carrying out the training of the model. The data set of the Sicilia region provided an independent data set for testing the model. The main characteristics of the two data sets are reported in Table 1. It is worth noting that the proportion of positive cases of varicella is different in the two databases. Interpreting differences in prevalence between regions is beyond the purpose of this study; nevertheless, given the smaller prevalence, there is an expected lower positive predictive value (PPV) and a higher negative predictive value (NPV) on the test set.

The Pedianet source data includes five different tables. In Table 2, we report a short description of them.

**Table 1:** Main characteristics used for the train (Veneto) and test (Sicilia) data sets.

Characteristic	Train	Test
Database	Pedianet	Pedianet
Language	Italian	Italian
Italian Region	Veneto	Sicilia
Date span	January 2, 2004-December 31, 2014	January 7, 2004-December 30, 2014
Records, n	1,230,355	569,926
Children, n	7631	2347
Pediatricians, n	46	13
Positive cases, n (%)	3481 (45.6%)	128 (5.4%)

**Table 2:** Tables used from the Pedianet database.

Table topic	Content	Type of data	Example
Accessing	Reasons for accessing the pediatrician and diagnoses	Free text (including codes)	Ritardo di crescita <783.4>
Diaries	Pediatrician’s free-text diaries	Free text	DIBASE OS GTT 10ML 10000UI/ML n° conf. 2\r\n per Visita di controllo e di follow up\r\n\r\n
Hospitalizations	Details on hospital admissions, diagnoses, and length of stays	Free text	Divisione di pediatria Tosse, difficolta' respiratoria e di alimentazione
SOAP^a^	Symptoms, objectivity, diagnosis, or prescriptions	Free text (including codes)	*SOAP*^b^: “P”, *SOAP_code*: “77469”, *SOAP_text*: “visita otorinolaringoiatrica<89.7>”
Specialistic visits	Visit type and its diagnosis	Free text including (codes)	*codice_visitaSP*: “89.01”, *visita*: “ecografia anche sec. Graaf per screening”, *diagnosi*: “problemi della vista <V41.0>”

^a^SOAP: symptoms, objectivity, diagnosis, or prescriptions.

^b^For tables with multiple fields, field names are reported in italics.

All the tables can be linked at the individual level (ie, each row of all the tables contains the fields for reporting information on dates, the assisting pediatrician’s anonymous identifier, and the patients’ anonymous identifier, which constitutes the linking key).

### Case Definition

The case definition comes directly from the gold standard provided, and the training set for machine learning was created using those dichotomous labels (ie, 0=noncase, that is not a varicella case; and 1=case, that is a varicella case).

### Training and Test Sets for Machine Learning

Linking by patient ID, pediatrician ID, and reporting date, we merged the five tables into a single table consisting of several entries, each of which represents a visit or evaluation of a patient carried out by a pediatrician on a specific day. At this step, the information (excluding patient ID, pediatrician ID, and reporting date) is contained in 15 columns containing free text mixed with coded text, which was considered by us as free text as well. Finally, all remaining columns of the table were merged into a single corpus (ie, a body of text). This process was applied to train the models on 1,230,355 entries (database of the Veneto region) and to test them on 569,926 entries (database of the Sicily region) separately.

### Preprocessing

Text analysis by a computer program is possible only after establishing a way to convert text (ie, readable to humans) into numbers (ie, readable to computers). This process is called preprocessing, and it is the first [23] and probably the most important step in data mining [24]. To process the corpus of Pedianet EHRs included in the training set, we used the following strategy. First, we converted all fields in a text type; lowered the content; and cleared it of symbols, punctuation, numbers, and extra white spaces. Second, we stemmed the words (ie, reducing them to their basic form, or “root”), which is recognized as one of the most important procedures to perform [25], and constructed 2-gram tokens, which has been shown to be the optimal rank for gram tokenization [26]. Third, we removed all the (stemmed) stop words (ie, common and nonmeaningful words such as articles or conjunctions) from the set of tokens as well as all bigrams containing any of them. We chose this strategy after exploring different approaches described in [27]. Fourth, we created the document-term matrix (DTM) as a patient-token matrix. To consider both the importance of the tokens within a patient (ie, one row of the DTM) and its discrimination power between patients’ records (ie, the rows of the DTM), we computed the TF-iDF (term frequencies–inverse document frequencies) weights. TF-iDF weights help to adjust for the presence of words that are more frequent but less meaningful [28]. TF-iDF-ij entry is equal to the product of the frequency of the j-th token in the i-th document by the logarithm of the inverse of the number of documents that contain that token (ie, the more frequent a word appears in a document the more its weight rises for that document), and the more documents that contain the j-th token, the more the weight shrinks across all the documents [29]. In the initial DTM there were 1,871,532 tokens that appear at least once, with a nonsparse/sparse entries ratio of (18,951,304/14,262,709,388). We decided to reduce it to achieve a maximum of 99% overall sparsity. Filtering out the tokens that do not appear in at least 1% of the documents had reduced it down to 94% (ie, 29,096 tokens that appear at least once for a nonsparse/sparse entries ratio of 13,140,370/208,891,206). The choice of a 99% level of sparsity was a tradeoff between the need to retain as many tokens as possible and the computational effort.

The corpus of Pedianet EHRs comprised in the test set went through the same text preprocessing strategy in the same order, and then the DTM was created with the initial TF weighing scheme. Furthermore, it was adapted with the same tokens retained in the training phase (ie, adding the missing tokens, weighting them as zero, and removing the ones not included in the training DTM) and was finally reweighted with the TF-iDF weighing scheme with the same retained iDF weights of the corresponding training DTM, which were retained when applied to the whole training data set. Those are necessary steps to guarantee that the two feature spaces are the same and that the models trained can be evaluated on the test set.

### Machine Learning Techniques

Enhancements of GLMs for carrying out text mining on EHRs have been proposed in the form of the lasso and GLMNet [16], multinomial logistic regression (MAXENT), and the boosting approach (LogitBoost) [16].

GLMNet is a regularized regression method that linearly combines the L1 and L2 penalties of the lasso and ridge methods applied in synergy with a link function and a variance function to overcome linear model limitations (eg, the constant variability among the mean and the normality of the data). The link function selected was the binomial (ie, the model fit a regularized logistic regression model for the log odds), while the amount of regularization was automatically selected by the algorithm through an exploration of 100 values between the minimum value that reduced all the coefficients to zero and its 0.01 fraction.

MAXENT is an implementation of (multinomial) logistic regression aimed at minimizing the memory load on large data sets in R (R Foundation for Statistical Computing) and is primarily designed to work with the sparse DTM provided by the R package [30]. It has been proven to provide results mathematically equivalent to a GLM with a Poisson link function [31].

Boosting is a general approach for improving the predictive capability of any given learning algorithm. We used the adaptations of Tuszynski [32] to the original algorithm, (ie, LogitBoost [33,34]), which is aimed at making the entire process more efficient while applying it on large data sets. The standard boosting technique [34] is applied to the sequential use of a decision stump classification algorithm as a weak learner (ie, a single binary decision tree). The number of stumps considered is the same as the columns provided in the training set.

Those techniques are chosen among computationally treatable algorithms for use with large data sets [30]. GLMNet and MAXENT represent classical benchmark approaches to linear and logistic classification, respectively, in a manner that differs from LogitBoost, which is a modern boosted tree-based machine learning approach [35,36]. Moreover, LogitBoost generalizes the classical logistic models by fitting a logistic model at each node [37] and shows an alternative point of view with regards to models such as the GLMs, for which the structure of the learner must be chosen a priori [38].

### Training and Testing

We addressed the issue of internal validation by performing cross-validation on the training set comprising records from the Veneto region. We dealt with external validation by accessing a truly external sample of Pedianet EHRs from another Italian region, Sicily. This accomplishes two tasks: preserving precision in the training phase and complementing study findings with external validation results using data that were not available when the predictive tool was developed.

We used a 5-fold cross-validation approach to validate each of the three MLTs on the DTM with the corresponding (by row) “case/non-case” attached labels. All MLTs were simultaneously fitted on the same set of folds to ensure a proper comparison between techniques. Values of k=10 or k=5 (especially for large data sets) have been shown empirically to yield acceptable (in terms of bias-variance trade-off) error rates [39,40]. Thus, the choice of 5-folds was driven by the computational complexity, the fewer folds, the less complexity.

As measures of performance, we calculated point estimates and 95% CIs for the following.

- PPV or Precision: 
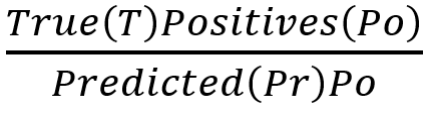
, that is the fraction of positively identified cases that are true positives
- NPV: 
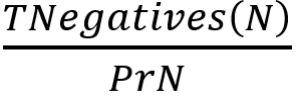
, that is the fraction of positively identified noncases that are true negatives
- Sensitivity or Recall: 
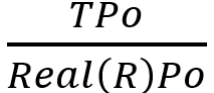
, that is the true positive rate
- Specificity: 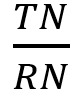, that is the true negative rate
- F score: 
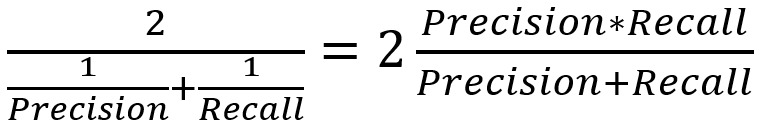
, the harmonic mean of the PPV (Precision) and Sensitivity (Recall)

The Gwet agreement coefficient 1 (AC1) statistics of agreement [41,42] between the techniques were computed and reported, along with their corresponding 95% CIs. Given that A=the number of times both models classify a record as noncase, D=the number of times both models classify a record as a case, and N=the total sample size, then 
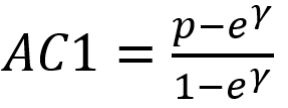
, where 
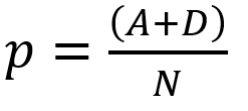
, and *e*^γ^ is the agreement probability by chance and is equal to 2*q* (1 – *q*), where 
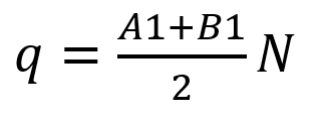
; A1 is the number of records classified as noncase by model 1, and B1 is the number of records classified as noncase by model 2. AC1 has been used given its propensity to be weakly affected by marginal probability, and therefore it was chosen to manage unbalanced data [43].

All the analyses were implemented in the R system [44] with the computing facilities of the Unit of Biostatistics, Epidemiology and Public Health. The R packages used were: *SnowballC* (to stem the words) and *RWeka* (to create n-grams) for the preprocessing step; *Matrix* and *SparseM* to manage sparse matrices; *GLMNet*, *MAXENT,* and *caTools* for the GLMNet, MAXENT, and LogitBoost MLT implementation; *caret* to create and evaluate the cross-validation folds; *ROCR* to estimate the performance; and the *tidyverse* bundle of packages for data management, functional programming, and plots. A git repository of the analysis code is available [45].

## Results

**Figure 1:**
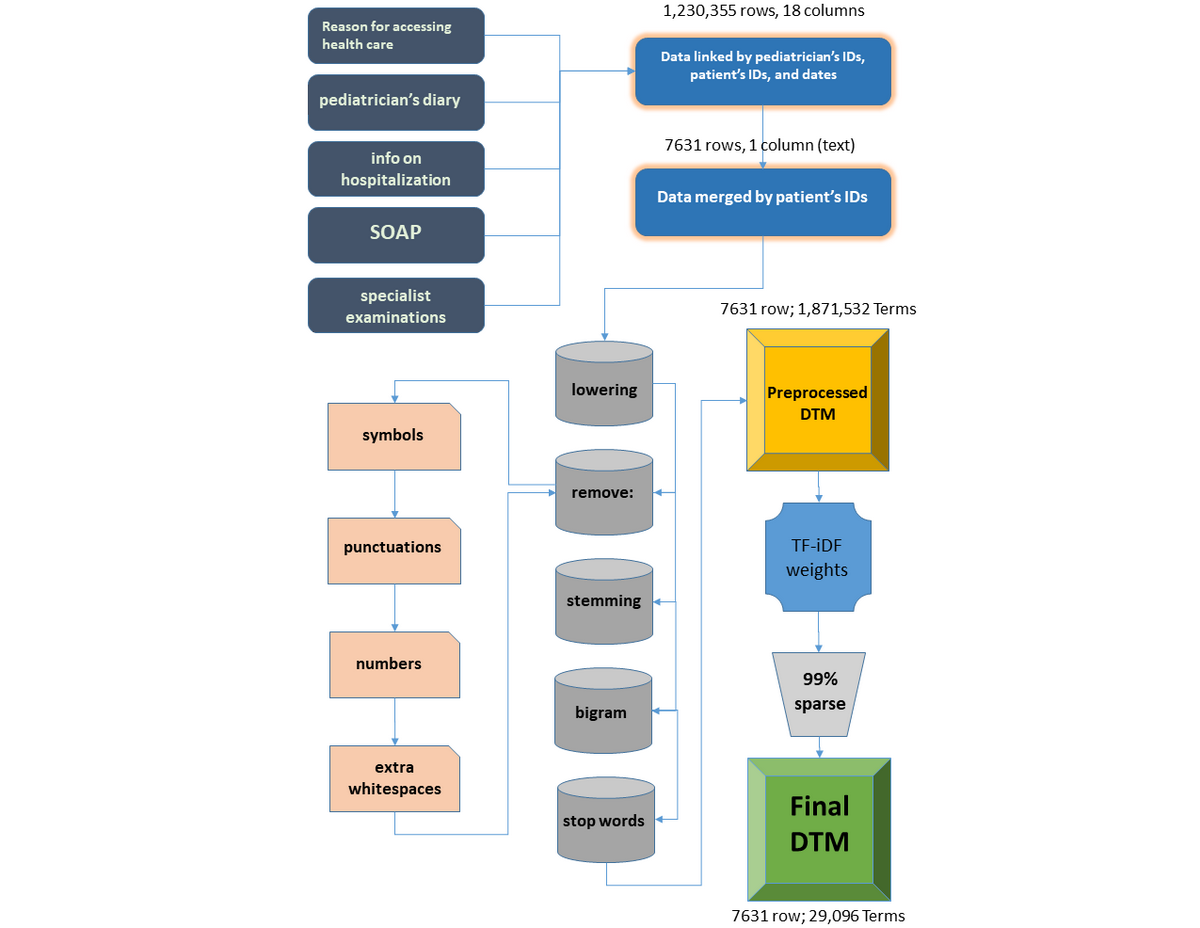
Flowchart from the acquisition of the five tables containing the electronic health records (dark gray) in the training set that were merged into a single table (dark blue); preprocessed (gray) with the specification of what was removed (pink) prior to the creation of the document-term matrix (DTM) (yellow); the computation of the weights (light blue); the dimensionality reduction, that is the reduction of the terms used (light gray), and the final DTM used (green). DTM: document-term matrix; SOAP: symptoms, objectivity, diagnosis, or prescriptions; TF-iDF: term frequencies–inverse document frequencies.

The flow chart, from data acquisition to preprocessing, is shown in Figure 1. In the training set, 29,096 initial terms out of 1,871,532 were retained by the sparsity reduction step. Boosting significantly outperforms all other MLTs on the training set, with the highest *F* score and PPV. The GLMNet predictor delivered a superior *F* score and greater PPV compared to MAXENT (Table 3). The same results held on the test set (Table 4) and agreement between MLT predictions on the training set was good as measured by AC1 statistics (Table 5).

**Table 3:** Performance on the training set of the three machine learning techniques using a 5-fold cross-validation method.

Technique	Sensitivity, mean (95% CI)	PPV^a^, mean (95% CI)	NPV^b^, mean (95% CI)	Specificity, mean (95% CI)	*F* score, mean (95% CI)
GLMNet^c^	80.2 (77.7-82.7)	73.2 (70.9-75.6)	90.9 (89.6-92.2)	87.1 (85.6-88.7)	76.5 (75.6-77.5)
MAXENT^d^	68.8 (66.8-70.7)	66.0 (62.5-69.5)	86.1 (85.2-86.9)	84.5 (82.7-86.3)	67.4 (64.7-70.0)
Boosting	86.6 (82.1-91.1)	95.8 (93.2-98.5)	94.4 (92.4-96.3)	98.3 (97.0-99.6)	90.9 (89.7-92.1)

^a^PPV: positive predicative value.

^b^NPV: negative predicative value.

^c^GLMNet: elastic-net regularized generalized linear model.

^d^MAXENT: maximum entropy.

**Table 4:** Performance on the test set of the three machine learning techniques under consideration.

Technique	Sensitivity, mean (95% CI)	PPV^a^, mean (95% CI)	NPV^b^, mean (95% CI)	Specificity, mean (95% CI)	*F* score, mean (95% CI)
GLMNet^c^	72.3 (66.4-78.1)	24.5 (21.0-28.0)	98.3 (97.9-98.6)	87.4 (85.4-89.5)	36.5 (32.2-40.8)
MAXENT^d^	74.8 (62.2-87.5)	11.0 (9.5-12.5)	98.0 (97.3-98.6)	65.5 (54.7-76.2)	19.1 (17.2-20.9)
Boosting	79.2 (69.7-88.7)	63.1 (42.7-83.5)	98.8 (98.3-99.3)	96.9 (94.2-99.6)	68.5 (59.3-77.7)

^a^PPV: positive predicative value.

^b^NPV: negative predicative value.

^c^GLMNet: elastic-net regularized generalized linear model.

^d^MAXENT: maximum entropy.

**Table 5:** Agreement between elastic-net regularized generalized linear model, maximum entropy, and boosting using 5-fold cross-validation.

Technique	Wrongly agree^a^, n	Correctly agree^b^, n	Disagree^c^, n	Gwet AC1^d,e^ (95% CI)
GLMNet^f^ vs MAXENT^g^	669	5609	1353	0.68 (0.67-0.70)
GLMNet vs boosting	195	6269	1146	0.74 (0.72-0.75)
MAXENT vs boosting	224	5895	1491	0.66 (0.65-0.68)

^a^The “Wrongly Agree” column refers to the number of records misclassified by both techniques.

^b^The “Correctly Agree” column states the number of records correctly classified by both techniques.

^c^The “Disagree” column lists the number of records for which the techniques disagree in the classification.

^d^AC1: agreement coefficient 1.

^e^Gwet AC1 represents the index of agreement between the identified techniques. Legend for AC1 is: AC1<0=disagreement; AC1 0.00-0.40=poor; AC1 0.41-0.60=discrete; AC1 0.61-0.80=good; AC1 0.81-1.00=optimal.

^f^GLMNet: elastic-net regularized generalized linear model.

^g^MAXENT: maximum entropy.

With the aim to analyze the most relevant errors, we explored if any records were wrongly classified by all the techniques. There were 3 records: 1 wrongly classified as positive and 2 wrongly classified as negatives by all the MLTs.

## Discussion

### Principal Findings

The application of MLTs to EHRs constitutes the analytical component of an emerging research paradigm that rests on the capture and preprocessing of massive amounts of clinical data to gain clinical insights and ideally to complement the decision-making process at different levels, from individual treatment to definition of national public health policies. As acknowledged by others [46], the development and application of big data analysis methods on EHRs may help create a continually learning health care system [47].

This study trains and compares three different machine learning approaches towards infectious disease detection at the population level based on clinical data collected in primary care EHRs. In line with the recommended paradigm for model validation [39], the MLTs’ performance underwent internal validation through cross-validation and external validation on an independent set of EHRs.

The predictive capabilities of the developed MLTs are promising even if quite different from each other (eg, validation *F* scores range from 67%-91% and test *F* scores range from 19%-69%). Findings on the better performance reached by LogitBoost are in line with recent evidence that shows an improvement in general classification problems moving from MAXENT algorithms to LogitBoost-based ones [48]. LogitBoost is thus confirmed to be a useful technique for solving health-related classification problems [34].

Only three records were wrongly classified by all the models. The first one was wrongly classified as positive probably because the text entry was “vaccini:varicella e mpr” (ie, vaccine: varicella and mpr), and after the preprocessing, the bigram “vaccin varicell” was removed because the TFiDF weight was low. Thus the relationship between varicella and vaccine was lost and remained only the token “varicell”.

The other two records were wrongly classified as negative. For one of them, the misclassification was probably due to an issue in the tokenization. In fact, an anomalous sequence of dashes (“-”) and blanks lead to the token “- varicella”, which was removed from the feature space, leaving no reference to the disease. The second negative misclassified record referred to a child who was vaccinated for measles, mumps, rubella, and varicella (quadrivalent vaccine). The pediatrician wrote “vaccinazione morbillo parotite rosolia varicella” (ie, vaccination, measles, mumps, rubella, varicella). The bigram “rosol varicell” (ie, “rubell varicell”) was weighted 0.361 and, hence, was retained in the feature space, and was considered by all the MLTs a pattern of noninfection.

The strength of tree-based models such as LogitBoost also lies in their high scalability. In fact, their computational complexity (ie, the asymptotical time needed for a complete run) grows linearly with the sample size and quadratically with the number of features used (ie, the number of tokens considered) [37]. Assuming that the richness of the pediatric EHRs’ vocabulary is limited (ie, the number of tokens reaches a plateau as data accumulates over time) an increase in computational time will only depend linearly on the number of patients.

Any attempt to use EHRs to identify patients with a specific disease would depend on the algorithm, the database, the language, and the true prevalence of the disease. As to the generalization of these models to other contexts, we hypothesize that they could also be successfully applied in public health systems with EHR charting in other languages [49].

We acknowledge that one metric (ie, sensitivity, specificity, PPV, or NPV) may be more important than another, depending on the intended use of the classification algorithm. Thus, the LogitBoost model is adequate for ascertaining varicella cases, with a preference for case identification with good sensitivity and excellent specificity.

If the aim of using MLTs is to help create a gold standard for databases, the limited agreement between the MLTs reported in Table 5 suggests that these classification algorithms are not reliable as a set of annotators.

### Limitations

Some limitations must be acknowledged. First, it is acknowledged that text preprocessing is a crucial step. The way to convert free text into numbers and numbers into features is an essential step of the process and has one of the biggest impacts on the results [24]. For the same reason as before, we decided to follow a standard preprocessing procedure without searching for the best one to obtain results that are, at most, independent of human tuning.

Second, we set the number of boosting iterations as the same number of features considered. This is suboptimal in computational time because the same performance can be reached with fewer iterations [37]. Nevertheless, we aimed to reach an upper-bound value for the performance estimated in an optimal situation.

Third, the large difference in disease prevalence between the training and the validation data set should be noted. The boosting approach seems to deal with this issue in a satisfactory way, but a potential impact on model prediction could not be excluded.

### Conclusions

Given their promising performance in identifying varicella cases, LogitBoost, and MLTs in general, could be effectively used for large-scale surveillance, minimizing time and cost in a scalable and reproducible manner.

## Abbreviations

AC1: agreement coefficient 1
DTM: document-term matrix
EHR: electronic health report
GLM: generalized linear model
GLMNet: elastic-net regularized generalized linear model
MAXENT: maximum entropy
MLT: machine learning technique
NPV: negative predicative value
PPV: positive predicative value
TF-iDF: term frequencies–inverse document frequencies

## References

[1] Magill SS, Dumyati G, Ray SM, Fridkin SK (2015). Evaluating epidemiology and improving surveillance of infections associated with health care, United States. Emerg Infect Dis.

[2] Lloyd-Smith JO, Funk S, McLean AR, Riley S, Wood JL (2015). Nine challenges in modelling the emergence of novel pathogens. Epidemics.

[3] Sutherland SM, Kaelber DC, Downing NL, Goel VV, Longhurst CA (2016). Electronic health record-enabled research in children using the electronic health record for clinical discovery. Pediatr Clin North Am.

[4] Baracco G, Eisert S, Saavedra S, Hirsch P, Marin M, Ortega-Sanchez I (2015). Clinical and economic impact of various strategies for varicella immunity screening and vaccination of health care personnel. Am J Infect Control.

[5] Damm O, Ultsch B, Horn J, Mikolajczyk RT, Greiner W, Wichmann O (2015). Systematic review of models assessing the economic value of routine varicella and herpes zoster vaccination in high-income countries. BMC Public Health.

[6] Kawai K, Gebremeskel BG, Acosta CJ (2014). Systematic review of incidence and complications of herpes zoster: towards a global perspective. BMJ Open.

[7] Pierik JG, Gumbs PD, Fortanier SA, Van Steenwijk PC, Postma MJ (2012). Epidemiological characteristics and societal burden of varicella zoster virus in the Netherlands. BMC Infect Dis.

[8] Jensen PB, Jensen LJ, Brunak S (2012). Mining electronic health records: towards better research applications and clinical care. Nat Rev Genet.

[9] Afzal Z, Schuemie MJ, van Blijderveen JC, Sen EF, Sturkenboom MC, Kors JA (2013). Improving sensitivity of machine learning methods for automated case identification from free-text electronic medical records. BMC Med Inform Decis Mak.

[10] Wang Z, Shah AD, Tate AR, Denaxas S, Shawe-Taylor J, Hemingway H (2012). Extracting diagnoses and investigation results from unstructured text in electronic health records by semi-supervised machine learning. PLoS One.

[11] Kavuluru R, Rios A, Lu Y (2015). An empirical evaluation of supervised learning approaches in assigning diagnosis codes to electronic medical records. Artif Intell Med.

[12] Ford E, Carroll JA, Smith HE, Scott D, Cassell JA (2016). Extracting information from the text of electronic medical records to improve case detection: a systematic review. J Am Med Inform Assoc.

[13] Zheng T, Xie W, Xu L, He X, Zhang Y, You M, Yang G, Chen Y (2017). A machine learning-based framework to identify type 2 diabetes through electronic health records. Int J Med Inform.

[14] Wu P-Y, Cheng C-W, Kaddi CD, Venugopalan J, Hoffman R, Wang MD (2017). -Omic and electronic health record big data analytics for precision medicine. IEEE Trans Biomed Eng.

[15] Friedman J, Hastie T, Tibshirani R (2010). Regularization paths for generalized linear models via coordinate descent. J Stat Softw.

[16] Friedman J, Hastie T, Tibshirani R (2000). Additive logistic regression: a statistical view of boosting (With discussion and a rejoinder by the authors). Ann Statist.

[17] Mani S, Chen Y, Arlinghaus L, Li X, Chakravarthy A, Bhave S, Welch E, Levy M, Yankeelov TE (2011). Early prediction of the response of breast tumors to neoadjuvant chemotherapy using quantitative MRI and machine learning. AMIA Annu Symp Proc.

[18] Pedianet.

[19] Nicolosi A, Sturkenboom M, Mannino S, Arpinelli F, Cantarutti L, Giaquinto C (2003). The incidence of varicella: correction of a common error. Epidemiology.

[20] Nicolosi A, Sturkenboom M, Mannino S, Arpinelli F, Cantarutti L, Giaquinto C (2003). The incidence of varicella: correction of a common error. Epidemiology.

[21] Cantarutti A, Donà D, Visentin F, Borgia E, Scamarcia A, Cantarutti L, Peruzzi E, Egan C, Villa M, Giaquinto C, Pedianet (2015). Epidemiology of frequently occurring skin diseases in Italian children from 2006 to 2012: a retrospective, population-based study. Pediatr Dermatol.

[22] Donà D, Mozzo E, Scamarcia A, Picelli G, Villa M, Cantarutti L, Giaquinto C (2016). Community-acquired rotavirus gastroenteritis compared with adenovirus and norovirus gastroenteritis in Italian children: a Pedianet study. Int J Pediatr.

[23] Sebastiani F (2002). Machine learning in automated text categorization. ACM Comput Surv.

[24] Denny MJ, Spirling A (2018). Text preprocessing for unsupervised learning: why it matters, when it misleads, and what to do about it. Polit Anal.

[25] Liu M, Hu Y, Tang B (2014). Role of text mining in early identification of potential drug safety issues. Methods Mol Biol.

[26] Marafino B, Davies J, Bardach N, Dean M, Dudley RA (2014). N-gram support vector machines for scalable procedure and diagnosis classification, with applications to clinical free text data from the intensive care unit. J Am Med Inform Assoc.

[27] Gregori D, Paola B, Soriani N, Baldi I, Lanera C (2016). Maximizing text mining performance: the impact of pre-processing. JSM Proceedings, Section on Statistical Learning and Data Science.

[28] Wu HC, Luk RWP, Wong KF, Kwok KL (2008). Interpreting TF-IDF term weights as making relevance decisions. ACM Trans Inf Syst.

[29] Goodall CR (1999). Data mining of massive datasets in healthcare. Journal of Computational and Graphical Statistics.

[30] Jurka T (2012). maxent: an R package for low-memory multinomial logistic regression with support for semi-automated text classification. The R Journal.

[31] Renner IW, Warton DI (2013). Equivalence of MAXENT and Poisson point process models for species distribution modeling in ecology. Biometrics.

[32] Tuszynski J (2019). R-project.

[33] Dettling M, Bühlmann P (2003). Boosting for tumor classification with gene expression data. Bioinformatics.

[34] Freund Y, Schapire RE (1996). Experiments with a new boosting algorithm. https://cseweb.ucsd.edu/~yfreund/papers/boostingexperiments.pdf.

[35] Boughorbel S, Al-Ali R, Elkum N (2016). Model comparison for breast cancer prognosis based on clinical data. PLoS One.

[36] Andrews P, Sleeman D, Statham P, McQuatt A, Corruble V, Jones P, Howells T, Macmillan CSA (2002). Predicting recovery in patients suffering from traumatic brain injury by using admission variables and physiological data: a comparison between decision tree analysis and logistic regression. J Neurosurg.

[37] Landwehr N, Hall M, Frank E (2005). Logistic model trees. Mach Learn.

[38] Abeare S (2009). LSU Master's Theses.

[39] Hastie T, Tibshirani R, Friedman J (2009). The Elements Of Statistical Learning.

[40] Borra S, Di Ciaccio A (2010). Measuring the prediction error. A comparison of cross-validation, bootstrap and covariance penalty methods. Computational Statistics & Data Analysis.

[41] Gwet Kl (2014). Handbook Of Inter-rater Reliability: The Definitive Guide To Measuring The Extent Of Agreement Among Raters.

[42] Wongpakaran N, Wongpakaran T, Wedding D, Gwet KL (2013). A comparison of Cohen's Kappa and Gwet's AC1 when calculating inter-rater reliability coefficients: a study conducted with personality disorder samples. BMC Med Res Methodol.

[43] Zec S, Soriani N, Comoretto R, Baldi I (2017). High agreement and high prevalence: the paradox of Cohen's kappa. Open Nurs J.

[44] (2016). R Foundation for Statistical Computing.

[45] GitHub.

[46] Ross M, Wei W, Ohno-Machado L (2014). "Big data" and the electronic health record. Yearb Med Inform.

[47] Wiens J, Shenoy ES (2018). Machine learning for healthcare: on the verge of a major shift in healthcare epidemiology. Clin Infect Dis.

[48] Xing C, Geng X, Xue H (2016). Logistic boosting regression for label distribution learning. http://openaccess.thecvf.com/content_cvpr_2016/papers/Xing_Logistic_Boosting_Regression_CVPR_2016_paper.pdf.

[49] Lorenzoni G, Bressan S, Lanera C, Azzolina D, Da Dalt L, Gregori D (2019). Analysis of unstructured text-based data using machine learning techniques: the case of pediatric emergency department records in Nicaragua. Med Care Res Rev.

